# Supplementary material for: A Prospective Observational Study Analyzing the Diagnostic Value of Hepcidin-25 for Anemia in Patients with Inflammatory Bowel Diseases
Source: Int J Mol Sci. 2024 Mar 22;25(7):3564. doi: 10.3390/ijms25073564 (PMC11011637; doi:10.3390/ijms25073564)
Supplement: Supplementary file 1 [file ijms-25-03564-s001.zip › ijms-2877079-supplementary.pdf]

Prospective observational study analyzing diagnostic value of hepcidin-25 of anemia in patients with inflammatory bowel diseases

Affiliations:
Stanko Petrović, MD, PhD, *,† Dino Tarabar, MD, PhD,‡ Danica Ćujić, PhD,§ Dusica Stamenkovic, MD, PhD,*,¶ Marijana Petrović,MD,PhD,*, || Nemanja Rančić, MD,PhD,*, ** Vesna Subota, PhD biology, †† Nenad Perišić, MD, PhD,*, † and Mihailo Bezmarević, MD, PhD,*, ‡‡

From the
* Medical Faculty of the Military Medical Academy, University of Defence, Belgrade, Serbia;
†Clinic for Gastroenterology and Hepatology, Military Medical Academy, University of Defense, Belgrade, Serbia;
‡Clinic for Gastroenterology, University Hospital Center “Dr. Dragiša Mišović,” Belgrade, Serbia;
§Laboratory for Biology of Reproduction, Institute for Application of Nuclear Energy, University of Belgrade, Belgrade, Serbia;
¶ Department of Anesthesiology and Intensive Care, Military Medical Academy, Belgrade, Serbia;
||Clinic for nephrology, Military Medical Academy, Belgrade, Serbia;
**Centre for Clinical Pharmacology, Military Medical Academy, University of Defense, Belgrade, Serbia;
††Institute of Medical Biochemistry, Military Medical Academy, University of Defense, Belgrade, Serbia;
and
‡‡Department of Hepatobiliary and Pancreatic Surgery, Unit for Perioperative Nutrition, Clinic for General Surgery, Military Medical Academy, University of Defense, 11000 Belgrade, Serbia;

Address correspondence to: Stanko Petrović, MD, PhD, Medical Faculty of the Military Medical Academy, University of Defence, Belgrade, Serbia; Clinic for Gastroenterology and Hepatology, Military Medical Academy, University of Defense, Crnotravska 17, 11 050 Belgrade, Serbia (e-mail: stanko53p@gmail.com, Phone: +381 63 762 2212)

Contents

**Figure S1**	Median values of hepcidin-25 in the Group I and Group II compared to the Control group.	**3**
**Table S1**	Demographic characteristics of study population	**4**
**Table S2**	Results of multiplex analysis of cytokines in sera of patients with IBD. Significant difference in measured levels between Group I and Group II was observed solely for cytokine IL-13	**5**
**Table S3**	Serum levels of markers of platelet activation in IBD patients.	**7**

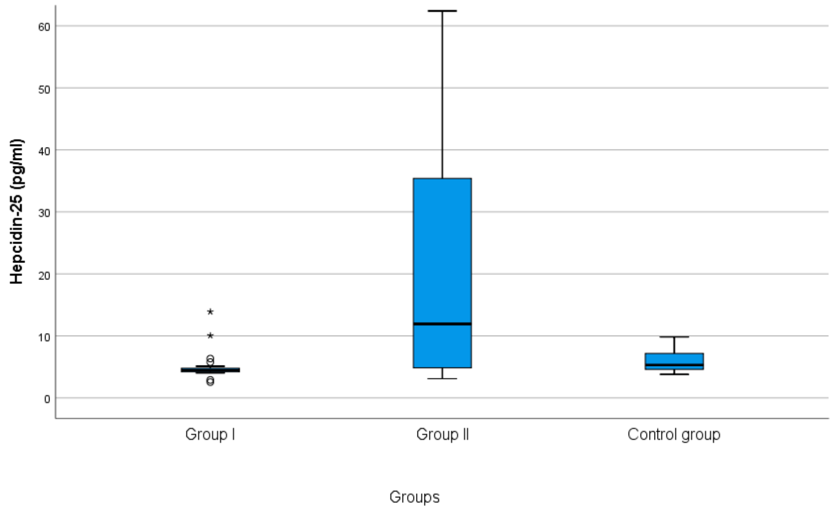

Figure S1. Median values of hepcidin-25 in the Group I and Group II compared to the Control group.

Supplementary Table S1. Demographic characteristics of study population.

	Group I			Group II			*P value*		
	**All (n=30)**	**UC (n=23)**	**CD (n=7)**	**All (n=32)**	**UC (n=26)**	**CD (n=6)**	**Group I vs Group II**	**UCI vs UCII**	**CDI vs CDII**
Age (year)	40.6±12.3	42.3±11.3	35.3±14.8	45.0±13.2	45.6±13.1	42.3±14.7	0.181	0.339	0.409
Sex (M/F)	15/15	11/12	4/3	21/11	18/8	3/3	0.323	0.128	1

Values are presented as mean(±SD) and n.
Abbreviations: UC I - I group, ulcerative colitis; UC II - II group, ulcerative colitis; CD I - I group, Crohn`s; CD II - II group, Crohn`s; M-male; F-female

Supplemantary Table S2. Results of multiplex analysis of cytokines in sera of patients with IBD. Significant difference in measured levels between Group I and Group II was observed solely for cytokine IL-13

	Groups and subgroups						p value		
	**Group I (n=30)**	**UC I (n=23)**	**CD I (n=7)**	**Group II (n=32)**	**UC II (n=26)**	**CD II (n=6)**	**Group I vs Group II**	**UC I vs UC II**	**CD I vs CD II**
IL-2 (pg/mL)	61.0 (32.0-85.0)	63.0 (37.0-94.0)	50.0 (30.0-82.0)	69.0 (45.2-109.5)	68.5 (41.0-93.8)	114.0 (50.2-286.8)	0.215	0.581	0.153
IL-4 (pg/mL)	0.0 (0.0-0.0)	0.0 (0.0-0.0)	0.0 (0.0-0.0)	0.0 (0.0-1.0)	0.0 (0.0-137.0)	0.0 (0.0-0.2)	0.172	0.288	0.28
IL-5 (pg/mL)	1.0 (0.0-161.8)	14.0 (0.0-167.0)	1.0 (0.0-23.0)	17.0 (0.0-156.5)	17.0 (0.0-168.2)	21.0 (0.0-83.8)	0.971	0.885	0.554
IL-6 (pg/mL)	67.0 (35.5-128.0)	72.0 (43.0-131.0)	41.0 (19.0-106.0)	85.0 (40.0-229.2)	85.0 (40.0-181.2)	179.0 (27.8-325.2)	0.494	0.912	0.317
IL-9 (pg/mL)	100.5 (39.8-173.5)	86.0 (41.0-150.0)	163.0 (0.0-282.0)	170.5 (44.7-264.5)	170.5 (52.2-263.0)	129.5 (30.0-280.5)	0.146	0.076	1
IL-10 (pg/mL)	104.5 (1.0-331.7)	194.0 (21.0-439.0)	1.0 (1.0-33.0)	129.5 (5.2-313.2)	100.5 (1.0-319.0)	224.5 (35.2-288.5)	1.000	0.356	0.084
IL-13 (pg/mL)	287.5 (97.2-440.2)	293.0 (125.0-440.0)	282.0 (14.00-441.0)	132.5 (19.0-211.8)	132.5 (16.0-234.2)	100.5 (16.5-236.5)	**<0.01**	**<0.01**	0.352
IL-17A (pg/mL)	83.5 (65.0-141.0)	87.0 (71.0-136.0)	65.0 (55.0-156.0)	102.0 (56.0-245.2)	92.5 (54.0-175.5)	261.0 (104.2-493.0)	0.662	0.554	0.116
IL-17F (pg/mL)	3.5 (0.0-138.8)	14.0 (0.0-174.0)	0.0 (0.0-17.0)	6.0 (0.0-85.5)	3.5 (0.0-90.0)	13.0 (1.0-96.5)	0.666	0.916	0.168
IL-21 (pg/mL)	164.5 (153.0-275.2)	172.0 (159.0-288.0)	159.0 (128.0-271.0)	186.0 (144.5-384.2)	183.0 (138.5-288.2)	361.5 (151.5-820.2)	0.386	0.904	0.116
IL-22 (pg/mL)	99.0	106.0	69.0	93.0	88.0	218.0	0.714	0.602	0.174

	(61.8-184.0)	(65.0-193.0)	(48.0-172.0)	(65.5-259.8)	(64.8-172.8)	(53.2-554.8)			
TNF-α (pg/mL)	188.0 (0.0-288.5)	212.0 (169.0-293.0)	0.0 (0.0-190.0)	232.0 (170.8-633.0)	227.5 (172.2-619.0)	249.0 (0.0-676.2)	0.120	0.314	0.229
IFN-γ (pg/mL)	6.0 (0.0-222.8)	27.0 (0.0-252.0)	0.0 (0.0-35.0)	62.5 (0.0-638.5)	81.0 (0.0-561.5)	44.5 (0.0-5468.8)	0.274	0.573	0.186

Abbreviations: UC I - I group, ulcerative colitis; UC II - II group, ulcerative colitis; CD I - I group, Crohn`s disease; CD II - II group, Crohn`s disease

Supplementary Table S3. Serum levels of markers of platelet activation in patients Inflammatory Bowel Disease (IBD)

	**Groups and subgroups**						**p value**		
	Group I (n=30)	UC I (n=23)	CD I (n=7)	Group II (n=32)	UC II (n=26)	CD II (n=6)	Group I vs Group II	UC I vs UC II	CD I vs CD II
sCD40L (pg/ml)	2158.5 (1816.7-3277.7)	2430.0 (1841.0-3274.0)	2078.0 (1740.0-3597.0)	1753.0 (975.2-3400.2)	1961.0 (1083.7-3256.7)	1564.5 (716.2-3647.0)	0.220	0.279	0.391
sPsel (ng/ml)	115.5 (54.2-165.0)	96.0 (57.0-174.0)	142.0 (44.0-162.0)	60.5 (43.5-93.0)	59.0 (39.5-88.5)	109.5 (47.2-172.5)	0.069	**0.047**	0.775

UC I - I group, ulcerative colitis; UC II - II group, ulcerative colitis; CD I - I group, Crohn`s disease; CD II - II group, Crohn`s disease
